# Telehealth-delivered cognitive rehabilitation for people with cognitive impairment as part of the post-COVID syndrome: protocol for a randomised controlled trial as part of the CICERO (Cognitive Impairment in Long COVID: Phenotyping and Rehabilitation) study

**DOI:** 10.1186/s13063-024-08554-3

**Published:** 2024-10-22

**Authors:** Martina Vanova, Aysha Mohamed Rafik Patel, Iona Scott, Gina Gilpin, Emily N. Manning, Charlotte Ash, Philippa Wittenberg, Jason Lim, Zoe Hoare, Rachel Evans, Nathan Bray, Christopher M. Kipps, Ciara Devine, Saliha Ahmed, Ross Dunne, Anna Koniotes, Catherine Warren, Dennis Chan, Aida Suarez-Gonzalez

**Affiliations:** 1 Dementia Research Centre, Faculty of Brain Sciences, UCL Queen Square Institute of Neurology, University College London, London, UK; 2 Institute of Cognitive Neuroscience, University College London, London, UK; 3 School of Applied Sciences, University of Brighton, Brighton, UK; 4 North Wales Medical School, Bangor University, Bangor, UK; 5 School of Health Sciences, Bangor University, Bangor, UK; 6 Department of Neurology, University Hospitals Southampton NHS Trust, Southampton, UK; 7 GM Dementia Research Centre, Greater Manchester Mental Health NHS Trust, Manchester, UK; 8 Geoffery Jefferson Brain Research Centre, University of Manchester, Manchester, UK; 9 Department of Neurology, University Hospitals Sussex NHS Trust, Brighton, UK

**Keywords:** COVID-19, Cognitive rehabilitation, Post-acute sequele of SARS-COV-2

## Abstract

**Background:**

Between 25 and 75% of people with persistent post-acute sequelae of SARS-CoV-2 infection (PASC) experience cognitive difficulties, compromising functional ability, quality of life, and activities of daily living, including work. Despite this significant morbidity, there is a paucity of interventions for this disorder that have undergone evaluation within a formal trial setting. Therefore, we have developed a cognitive rehabilitation programme, specifically designed to address the cognitive symptoms of PASC, notably impaired attention and processing speed, while also accounting for other PASC symptoms (fatigue, post-exertional malaise) that may aggravate the cognitive impairment. This study protocol outlines a randomised controlled trial (RCT) designed to evaluate the effectiveness of this programme compared to standard clinical care.

**Methods:**

This is a multi-centre, parallel-group, individually randomised controlled trial, comparing standard clinical care with and without cognitive rehabilitation. We will recruit 120 non-hospitalised adults (aged 30–60 years) from three NHS sites in England with a history of COVID-19 infection and cognitive impairment persisting more than 3 months after the acute infection. Participants will be randomised (1:1) to the intervention or control groups, with the latter represented as a provision of standard clinical care without cognitive rehabilitation. The cognitive rehabilitation programme consists of ten 1-hour sessions, delivered weekly. Outcomes will be collected at baseline, 3, and 6 months, with participant-defined goal-attainment scores, relating to functional goals, at 3 months as the primary outcome measure. Secondary outcomes will be cognitive function, measures of quality of life, social functioning, mental health, fatigue, sleep, post-exertional malaise, and social and health care service use. We will also evaluate the health-economic benefits of cognitive rehabilitation in this population.

**Discussion:**

Cognitive impairment in PASC is a major cause of functional disability with no effective treatment. Accordingly, we will undertake an RCT of cognitive rehabilitation, the protocol of which is published here. If this trial is successful in delivering improvements in trial outcomes, it will address a major unmet need relating to this emergent disorder, with a significant impact on affected individuals and the wider health economy.

**Trial registration:**

ClinicalTrials.gov NCT05731570. Registered on February 16, 2023

## Introduction

### Background and rationale {6a}

Persistent cognitive impairment is increasingly recognised as a major component of post-acute sequelae of SARS-CoV-2 infection (PASC) [1]. It is distinct from the cognitive sequelae of acute COVID-19 and other neurological disorders such as stroke and encephalitis. Estimates of the prevalence of cognitive impairment in PASC vary from 20 to 75% [1–3]. Cognitive impairment in PASC is characterised primarily by impairments in executive function, attention, speed of information processing [4], and working memory [3, 5–7], with the impairment independent of age, gender, or prior medical conditions [8]. The notion that cognitive impairment is a major and distinct aspect of PASC is reinforced by observations that the occurrence of cognitive impairment is unrelated to prior ITU admission and is not associated with fatigue, depression, or severity of acute inflammation [9]. These studies also show that cognitive impairment as part of PASC occurs even after milder infections, independent of comorbidities or stress reactions. The potential negative impact on working-age individuals, and in turn personal and national economies, is underscored by a UK study showing that dementia-range cognitive impairment was found in 26% of affected individuals, half of whom were working-age [10].

The loss in cognitive ability has major consequences for affected people, their families, and the wider economy given the problems caused regarding return to work. Moreover, its impact has been observed on overall well-being and quality of life [9–11], and regulation of emotions—symptoms of anxiety, depression, and mood swings [12]. Reduction in occupational and social activities like participating in hobbies, engaging in physical activities, and reduction in social interactions have been reported as potentially leading to social isolation [13], with an overall reduced ability to carry out daily tasks [14].

However, despite the major morbidity associated with this new disorder, to date, there are no evidence-based treatments for PASC cognitive impairment. Therefore, this represents a major unmet need in the management of PASC and there is an imperative to identify and test potential treatment options.

#### Cognitive rehabilitation

Cognitive rehabilitation represents one possible treatment. There is an extensive history of cognitive rehabilitation being successfully applied to other acquired cognitive disorders, notably, traumatic brain injury (TBI) and multiple sclerosis (MS) [15], whose profiles of cognitive impairment share similarities with that observed in PASC [16]. Cognitive rehabilitation has helped people with TBI to improve their attention, learning, and memory, and to develop compensatory activities. This has led to better performance in activities of daily living [17, 18]. Techniques like goal management training and external cueing can lead to improvement in executive functioning, better organisation and planning and long-term functional gains in real-world tasks [19]. In multiple sclerosis, cognitive impairment often involves reduced processing speed, memory, and executive dysfunctions that negatively impact the quality of life [20]. Cognitive rehabilitation using self-generation, repetition strategies, and functional aids have been found to improve memory, processing speed, and daily task performance in people with MS [21].

#### Telehealth-delivered cognitive rehabilitation

While traditional methods have involved lengthy face-to-face sessions, the requirement for social distancing during the COVID-19 pandemic necessitated a wholesale pivot towards remote provision of clinical care [22]. However, the potential utility of remotely applied cognitive rehabilitation in PASC is supported by precedents for this approach, with telehealth platforms having previously been used to deliver cognitive interventions in dementia [23] and MS [24]. While telehealth introduces some technical and logistical considerations [25], it confers notable advantages over traditional approaches that are especially relevant for this clinical population. Remote application helps remove barriers of geography and mobility that can limit access to in-person services. It has shown to be also a cost-effective way to deliver therapies and have a positive impact on quality of life [26] across patient and demographic groups [27]. Given that COVID-19 in the UK disproportionately affected ethnic minority populations [28], historically disadvantaged in access to health services, such issues are of particular salience. Furthermore, the fatigue that represents a major symptom of PASC might make it difficult for affected individuals to complete traditional face-to-face cognitive rehabilitation sessions, potentially compromising the treatment effect. Remote delivery of the treatment in shorter segments mitigates this risk in a way that has advantages both for the provider and the recipient of treatment.

This manuscript focuses on the study protocol for an RCT to test the effectiveness and cost-effectiveness of this rehabilitation programme to improve personally identified functional outcomes by people living with post-COVID cognitive impairment.

## Objectives {7}

This study aims to test the effectiveness of a cognitive rehabilitation programme (COVID-Rehab) in improving functional and cognitive outcomes in people living with cognitive impairment in PASC who did not require ICU admission at the time of infection, undertaken as part of the CICERO (Cognitive Impairment in Long COVID: Phenotyping and Rehabilitation) study. Guided by previous research evidence in the field of cognitive rehabilitation, we created a rehabilitation protocol, revised by our Patient and Public Involvement (PPI) group of experts by experience and an advisory group of neuropsychologist experts in cognitive rehabilitation. The neuropsychological intervention programme follows the NICE guidelines on stroke rehabilitation [29], the SIGN guidelines on brain injury rehabilitation [30], and evidence provided by recent systematic reviews [31, 32].

The research questions of this study are:

- Can cognitive rehabilitation improve the cognitive and functional outcomes of people presenting with cognitive impairment as part of post-COVID syndrome?
- What are the health-economic benefits of cognitive rehabilitation when applied to this patient population?

## Trial design {8}

This protocol has been registered in ClinicalTrials.gov (NCT05731570) and follows the Standard Protocol Items: Recommendations for Interventional Trials (SPIRIT 2013) [33] and will be reported according to the Consolidated Standards of Reporting Trials (CONSORT 2010) [34] reporting guidelines. To minimise the risk of unblinding and contamination of research participants and following the recommendations by Basu and colleagues [35], this protocol is being submitted during the final phase of the trial, but before the completion of data collection. This measure aims to reduce the likelihood of prospective participants deliberate biasing assessment outcomes and to prevent control group participants from gaining access to the details of the intervention administered to the intervention group. The Patient-Public Involvement (PPI) component of this study will be reported following the Guidance for Reporting Involvement of Patients and the Public (GRIPP2-SF) [36].

**Fig. 1:**
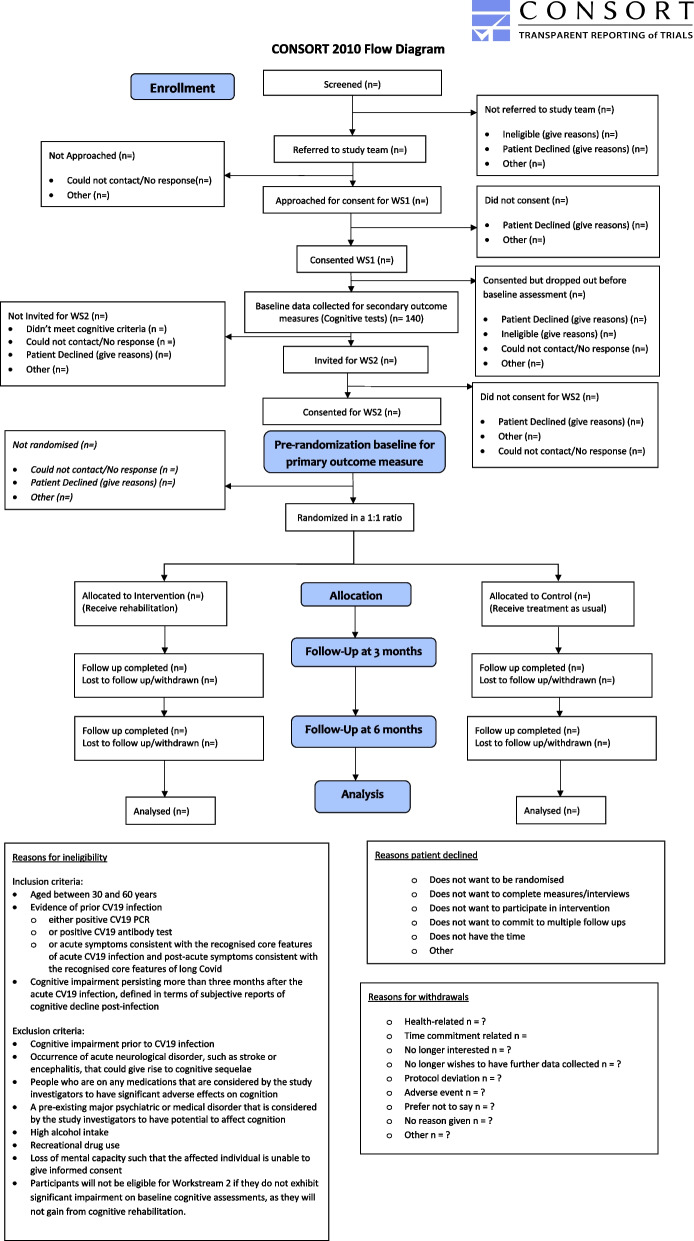
CONSORT 2010 flow diagram

This is the protocol for a multicentre individually randomised controlled trial with parallel groups and treatment allocation with a 1:1 ratio to active intervention and control arms. Outcome measures Data collection, management, and analysis will be collected at baseline, 3 and 6 months after randomisation. The study participant flow chart is shown in Fig. 1 and the RCT procedure is described below.

Participants allocated to the intervention group will receive ten sessions of cognitive rehabilitation delivered using telehealth. Those in the control group will receive treatment as usual (TAU), representing the standard clinical care they were receiving before entry into the study. Those in the control group will also receive cognitive rehabilitation educational resources at the end of the study. This is aimed at decreasing the attrition rate and meeting ethical demands in case the intervention has a beneficial effect. A subgroup of trial participants will be invited to take part in focus groups at the end of the study to improve our understanding of participants’ perceptions about barriers and facilitators for therapy uptake and recovery.

There is considerable evidence to support the hypothesis that people with cognitive dysfunction in PASC may positively respond to neuropsychological rehabilitation.

## Methods: participants, interventions, and outcomes

### Study setting {9}

Participants with cognitive impairment in PASC will be recruited from NHS memory clinics in Sussex, Southampton, and Greater Manchester in the UK.

### Eligibility criteria {10}

- Age between 30 and 60 years. This age range intends to minimise bias due to developmental factors and incomplete brain maturity and comorbidities prevalent in older populations (e.g. dementia) [37].
- Evidence of prior COVID-19 infection. This includes either positive COVID-19 polymerase chain reaction (PCR) test, antibody test, or acute symptoms consistent with the recognised core features of acute COVID-19 infection and post-acute symptoms consistent with the recognised core features of PASC.
- Self-reported cognitive impairment persisting more than 3 months after acute COVID-19 infection.
- Scores equal to or below 1 SD age-adjusted mean in at least two of the following cognitive domains: immediate memory, visuospatial memory, language, attention, delayed memory, information-processing speed, executive functioning, inhibition, and verbal fluency.

The exclusion criteria are as follows:

- Cognitive impairment prior to COVID-19 infection
- Occurrence of acute neurological disorder (e.g. stroke, encephalitis) with the potential to affect cognition
- Taking medication that is considered to have adverse effects on cognition
- A pre-existing major psychiatric or medical disorder that may affect cognition
- High alcohol intake
- Recreational drug use
- Inability to give informed consent due to loss of mental capacity

### Who will take informed consent? {26a}

**Fig. 2:**
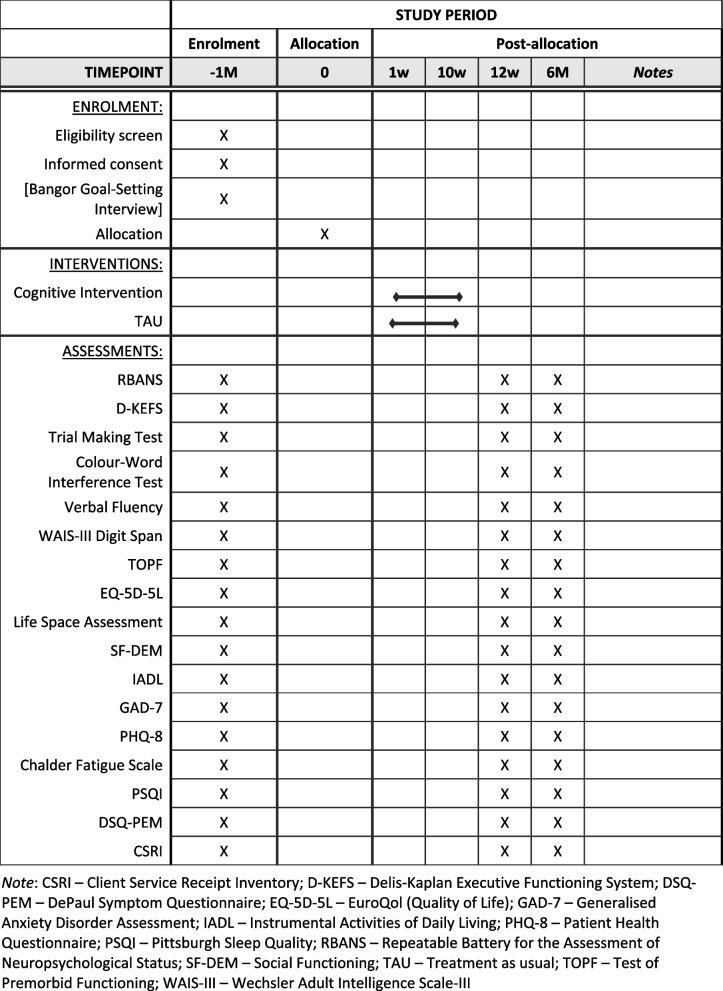
Standard Protocol Items: Recommendations for Interventional Trials (SPIRIT) figure. Items to be addressed in the clinical trial

Participants receive the information sheet about the study’s nature and procedures and give online informed consent to participate via the REDCap platform [38] (Fig. 2). Once consent is given, they receive a link to a survey to complete online demographic information and baseline secondary outcome measures on functioning and quality of life. Participants are then invited for in-person cognitive testing (secondary outcome measures). The eligibility for the RCT component of CICERO is assessed based on the cognitive scores. Eligible participants are invited to take part in the RCT, and online informed consent is obtained. The procedure is outlined in Fig. 1, following the CONSORT 2010 guidelines [34].

### Additional consent provisions for collection and use of participant data and biological specimens {26b}

Control group participants will be invited to attend an online workshop and a focus group, which will discuss strategies for neuropsychological rehabilitation of cognitive impairment in PASC. We will obtain online consent from participants to record these sessions for analysis purposes and to use de-identified quotations in publications.

## Interventions

### Explanation for the choice of comparators {6b}

#### Control group: Treatment as usual (TAU)

The control group will receive TAU, which is the standard of clinical care they were receiving on an individual basis prior to entry into the study.

### Intervention description {11a}

#### Experimental group: intervention

For the intervention programme, we will implement a collection of strategies informed by past work on the use of neuropsychological rehabilitation to alleviate cognitive deficits arising from TBI and MS. The final collection of strategies and their mode of implementation will be discussed and co-produced in collaboration with a group of people living with cognitive impairment in PASC (referred to as “experts by experience”—see “Patient-Public Involvement (PPI)” section). However, we anticipate that each therapy session will include, among others: training on restorative strategies to support the learning of new information, modelling of specific strategies and skills, improving the efficiency of existing strategies, and improvement or compensation of attentional and concentration disorders. Some of the strategies that will be implemented are goal management, chunking, pacing, environmental modifications, dual-task training, and multimodal encoding.

### Criteria for discontinuing or modifying allocated interventions {11b}

Suitable strategies adjusted to the individual needs will be selected for each individually identified goal. Participants will receive tasks to complete between sessions and apply in everyday situations. Strategies will be refined and adjusted over sessions as needed.

### Strategies to improve adherence to interventions {11c}

Participants will receive tasks to complete in between sessions and apply the strategies in everyday situations. Participants will complete a short online survey 2–3 times a week to monitor their progress and intervention adherence. Strategies will be refined and adjusted over sessions as needed.

#### Patient-Public Involvement (PPI)

The PPI lead, working alongside the study investigators, will help guarantee the investigator’s adherence to high-quality standards of PPI reporting as stated in the Guidance for Reporting Involvement of Patients and the Public (GRIPP) [36]. The PPI group for this study will consist of a group of four people living with cognitive symptoms of PASC. They will be identified with the help of the PPI lead through peer support networks and advocacy groups. PPI group meetings will take place every 3 months until the start of the RCT. We will collect feedback from the PPI group through minutes of group meetings and in the form of written notes and questionnaires and feedback or participation by email. The group will be trained at the start of the study on the method of participation (e.g. how to join by video meeting, what the rules of participation will be, how to provide feedback). The PPI team will extensively advise on the design and conduct of the intervention sessions (e.g. elements of the intervention and the best way to administer them), and on the dissemination of outcomes.

### Relevant concomitant care permitted or prohibited during the trial {11d}

No restrictions to concomitant care will be applied. This ensures that participants will receive necessary medical care during the study period. All concomitant medications and treatments will be recorded throughout the study to allow analysis of potential confounders to treatment. Participants will be advised to postpone participation in other interventional clinical trials until their participation in this study is concluded (6-month follow-up). This request aims to minimise potential confounding effects from other experimental interventions.

### Provisions for post-trial care {30}

At the end of the data collection, participants in the control group will be invited to an online workshop with a focus group where they will receive information about the study findings and about how to use, for their benefit, the rehabilitation strategies and techniques found successful in the intervention group. Anonymised feedback collected from these meetings will inform our creation of educational material and tutorials that will be made available on NHS websites.

## Outcomes {12}

### Demographics

Demographic information about age, date of birth, sex, gender, sexual orientation, ethnicity, language, education, COVID-19 diagnosis onset, occupation, and income will be collected via case report forms (online via REDCap) after consent to participate in the study is obtained.

### Primary outcome measure: Bangor Goal-Setting Interview (BGSI)

The main outcome measure of the cognitive intervention programme is goal achievement scores in three personalised goals identified by each participant at baseline, using the Bangor Goal-Setting Interview (BGSI) [39]. This is a manualised interview process to identify three SMART (specific, measurable, attainable, relevant, and time-bound) goals with each participant at baseline. The BGSI assesses each goal attainment and satisfaction on a scale of 1–10. Each goal is also scored on the same scale for (i) the participant’s readiness to change, (ii) perceived difficulty to achieve, and (iii) importance. The primary endpoint is the goal attainment score at 3 months post-randomisation. The mean goal attainment score between intervention and control groups, accounting for baseline scores, will be compared.

### Secondary outcome measures

The secondary measures will encompass a range of cognitive and functional measures, quality of life, health, and psychopathology symptoms, as described below. All secondary outcomes will be analysed at 3 and 6 months post-randomisation, with a comparison of mean scores between intervention and control groups taking into account baseline scores. Except for the DePaul Symptom Questionnaire—Post-Exertional Malaise (DSQ-PEM) [40] which is a binary (yes/no) outcome, the proportions of participants between groups will be compared on this measure.

#### Cognitive function

All participants will be tested using the following cognitive batteries: [1] The Repeatable Battery for the Assessment of Neuropsychological Status (RBANS) [41]—immediate memory, visuospatial memory, language, attention, delayed memory; [2] Delis–Kaplan Executive Functioning System (D-KEFS) [42] Trail Making Test—number sequencing, number-letter sequencing, Colour-Word Interference Test—colour naming, word naming, inhibition, inhibition/switching, Verbal Fluency Test—letter fluency, category fluency, category switching; [3] Wechsler Adult Intelligence Scale-III (WAIS-III) [43] Digit Span; [4] Test of Premorbid Functioning (TOPF) [44].

Participants will further complete the following digital tests: [1] The Long COVID Cognitive Assessment Battery (LCCAB) developed on the Gorilla platform [45], a variation of an established theory-based cognitive battery [46], evaluating processing speed, inhibitory control, attention, and working memory; [2] 4 Mountains Task, evaluating allocentric spatial memory [47]; and [3] Neotiv suite (https://www.neotiv.com/en) [48] of app-based cognitive tests, probing aspects of episodic memory, representing different processes and functions of the hippocampus and entorhinal cortex (pattern completion, scene recognition, and mnemonic discrimination).

#### Functional measures and quality of life

Additionally, the following measures assessing everyday functioning and quality of life will be administered: [1] Quality of Life (EQ-5D-5L) [49]—a standardised instrument for measuring generic health status; [2] Life Space Assessment [50]—a measure of functional mobility based on the distance reported in the past week; [3] Social Functioning (SF-DEM) [51]—self-assessment of social functioning; [4] Instrumental Activities of Daily Living (IADL) Scale [52]—assesses the ability to perform tasks required for independent living; [5] Generalised Anxiety Disorder Assessment (GAD-7) [53]—screening for severity of generalised anxiety disorder symptoms; [6] Patient Health Questionnaire (PHQ-8) [54]—screening for severity of depression symptoms; [7] Chalder Fatigue Scale [55]—assessment of physical and mental fatigue; [8] Pittsburgh Sleep Quality (PSQI) [56]—assesses sleep quality and disturbances; [9] DePaul Symptom Questionnaire—Post-Exertional Malaise (DSQ-PEM) [40]—measures the frequency and severity of post-exertional malaise symptoms; [10] Client Service Receipt Inventory (CSRI) [57]—a measure of service utilisation, income, accommodation, and cost-related variables.

This study will collect qualitative experiential feedback from participants through focus group interviews with a subpopulation of the study cohort. A subgroup of trial participants will be invited to take part in focus groups at the end of the study. This will improve our understanding of participants’ perceptions of barriers and facilitators for recovery and provide additional insights into the cognitive symptoms of PASC.

### Participant timeline {13}

Eligible participants receive an email with a booking link for the BGSI time slot via Qualtrics. The primary outcome measure (BGSI) takes place online via video call and afterwards, the participants are randomised. The randomisation outcome is announced to the participants via email. Those allocated to the intervention group will book their first session online. Primary and secondary outcome measures are collected in both groups at baseline, after 3 and 6 months post-randomisation. Figure 2 features a diagram outlining the participant timeline of enrolment, interventions, and assessments (including follow-ups) and follows the SPIRIT guidelines [33].

### Sample size {14}

In the absence of any prior work on this patient population that could inform power calculations for this study, results from the GREAT trial of cognitive rehabilitation in neurodegenerative disease using the same primary outcome (BGSI) and which indicated an effect size (Cohen’s *d*) of approximately 0.8 in a population with dementia were used as a guide [58]. However, we can expect a higher effect size in our RCT given that the GREAT trial involved people with a progressive neurodegenerative disorder, evidence for which is currently lacking in PASC. The baseline SD observed in the GREAT trial was 1.74. The variability in our sample will likely be larger. Thus, we can accommodate an increase in variability of approximately 65%. Assuming a minimal clinically important difference of 2 points (https://www.thecopm.ca/faq) [59] and a more conservative effect of 0.7, this would result in the measure SD of 2.86, approximately.

Based on these calculations, a sample of 88 participants (44:44) will be required to detect an effect of 0.7 on the BGSI at 3 months with 90% power at a 5% significance level. Incorporating an attrition of 25% will require recruitment and randomisation of 120 participants (60:60).

### Recruitment {15}

Participants will be recruited through NHS memory clinics in Sussex, Southampton, and Greater Manchester. Patients who have previously consented to be contacted for research purposes will be approached and given the Participant Information Sheet. University Hospitals Sussex NHS Foundation Trust will also recruit from Sussex Community NHS Foundation Trust. These additional recruitment sites will be established as Participant Identification Centres (PICs). The study will be promoted through various channels (website, newsletter, and social media platforms) and recruitment posters in clinics.

## Assignment of interventions: allocation

### Sequence generation {16a}

The randomisation procedure uses a dynamic data algorithm to protect against subversion [60]. The probability of allocation to each group is varied based on the information of those already randomised. This ensures the trial maintains a good balance to the allocation ratio of 1:1, both within the stratification variable (site) and overall, for the trial. Randomisation will be stratified by site.

### Concealment mechanism {16b}

The randomisation system will be set up, maintained, and monitored independently of the trial statistician or other trial staff by the North Wales Organisation for Randomised Trials in Health (NWORTH) Clinical Trials Unit.

### Implementation {16c}

When a participant has completed their baseline primary outcome measurement (BGSI) online, their details will be entered by the study primary investigator (PI) (namely ASG) into the secure online randomisation system. Details of the randomisation outcome will be emailed to the relevant researcher by the researcher conducting the interventions (namely MV).

## Assignment of interventions: blinding

### Who will be blinded {17a}

Due to the nature of the intervention, participants cannot be blinded to the allocation they receive. Researchers collecting the primary and secondary outcome measures at follow-ups 1 and 2 will be blinded. However, the allocation may become apparent during the participant’s follow-up assessment, as details about their goal performance are discussed. Data analysis will be completed blinded with adherence analysis being completed after the main analysis has been discussed and interpreted.

### Procedure for unblinding if needed {17b}

As participants are not blinded to allocation, there is no requirement for emergency unblinding for the study. Once all blinded analysis has been conducted, the statisticians will be officially unblinded following NWORTH standard operating procedures, and further analysis of intervention data will be conducted.

## Data collection and management

### Plans for assessment and collection of outcomes {18a}

All relevant data inputs will undergo full validation and verification by the trial team, and uncertainty will be considered within the sensitivity analysis.

### Plans to promote participant retention and complete follow-up {18b}

Control group participants will be invited to participate in a workshop to support their knowledge and coping strategies for PASC, which can increase retention. Data collection will continue for participants who withdraw from the intervention, for analysis purposes.

### Data management {19}

All participant data will be pseudonymised and stored securely on password-protected UCL servers. Identifiable information will be kept separately in the UCL Data Safe Haven with limited access. Participant codes will be used for analysis to maintain anonymity.

### Confidentiality {27}

Only clinical and research staff can access identifiable data to coordinate participation. Data transfers from third parties will use the Data Safe Haven portal.

### Plans for collection, laboratory evaluation, and storage of biological specimens for genetic or molecular analysis in this trial/future use {33}

Not applicable. No biological samples will be collected.

## Statistical methods

### Statistical methods for primary and secondary outcomes {20a}

The primary analysis will be completed per protocol at the 3-month endpoint using a linear mixed-effect model, adjusted for baseline scores, and randomising site (random effect). All treatment effect estimates will be presented with 95% confidence intervals. Analysis of continuous secondary outcomes will follow the same analysis model as the primary analysis where possible. Binary outcomes will be analysed using multi-level logistic regression. The aim will be to minimise missing data; however, predictors of missingness will be investigated using regression modelling and any variables found to be predictors will be considered for inclusion in the models. Multiple imputations will address missing scores where appropriate, and sensitivity analyses will be considered to assess the impact of these assumptions on the observed outcome. Exploratory analysis will be conducted to investigate the effect of phenotypes and comorbidities on the outcome and also the impact of adherence to the intervention. A full statistical analysis plan will be written and agreed upon by the investigators prior to the completion of data collection.

### Interim analyses {21b}

No interim analyses are planned. Due to the nature of the intervention, we do not plan to analyse either for safety or futility. Therefore, the power calculation has not accommodated for any interim analysis.

### Methods for additional analyses (e.g. subgroup analyses) {20b}

#### Economic evaluation

From a public sector, multi-agency perspective we will conduct an economic evaluation to determine the incremental cost-effectiveness of the COVID-Rehab intervention programme in comparison to TAU. We will examine two additional research questions during the economic evaluation:

1. What is the incremental cost-effectiveness of COVID-Rehab compared to TAU for the treatment of post-COVID cognitive impairment?
2. What is the cost per quality-adjusted life year (QALY) of COVID-Rehab compared to TAU for the treatment of post-COVID cognitive impairment, and does this fall below the NICE threshold of £20,000 to £30,000 per QALY?

####  Costing

CSRI data will be used to calculate participants’ utilisation of primary and secondary health and social care services over the follow-up period. National unit costs [61], routine hospital data, and consultation with clinical partners will be used to cost the intervention, TAU, and service use. Discounting of costs and outcomes will not be relevant as the follow-up period is less than 12 months.

#### Primary and secondary cost-effectiveness analyses

We will conduct our economic evaluation at the end of the 3-month follow-up period, based on the following components:

- Conduct a primary cost-utility analysis using the EQ-5D-5L as the measure of utility to generate a cost per QALY estimate.
- Conduct a secondary cost-effectiveness analysis using relevant trial outcomes (Bangor Goal-Setting Interview, GAD-7), and develop incremental cost-effectiveness ratios (ICERs) to express potential cost-effectiveness.
- Use bootstrapping to produce cost-effectiveness acceptability curves (CEACs) for comparison with the NICE ceiling of £20,000 to £30,000 per QALY in the UK.
- Conduct sensitivity analyses to investigate uncertainty in the data.

If neither treatment arm is found to have a significant effect on patient outcomes, we will present a cost-consequence analysis whereby the economic costs and outcome will be presented in a disaggregated manner.

#### Sensitivity analyses

We will use both deterministic and probabilistic sensitivity analysis to test the uncertainty of findings. Upon completion of the initial data analysis, we will determine the most appropriate approach to deterministic sensitivity analysis. We will consider whether to take a univariate or multivariate approach, where single or multiple parameters may be individually adjusted (within a given range of uncertainty) to test findings. As an example, we will examine the extent to which incrementally increasing or decreasing the cost of the intervention (within given confidence limits) impacts cost-effectiveness outcomes. Probabilistic sensitivity analysis will be used to assign a distribution of point estimates to each parameter and to extrapolate the model calculation. By running a number of replications (*n* = 5000), an ICER plane will be generated to illustrate the potential variation in cost-effectiveness based on altering basic assumptions about effectiveness and costs.

### Methods in analysis to handle protocol non-adherence and any statistical methods to handle missing data {20c}

Every effort will be taken to minimise missing data. If imputation (e.g. mean substitution) is part of the validated measure, it will be performed for measure scoring rules. Where there are no missing data rules for the measure, if the number of missing items on an outcome is 20% or less, then the missing value for the item will be substituted by the individual’s mean score for the remaining items on the scale [62]. If there are more than 20% missing items in the scale, the outcome measure will not be calculated for the participant at that time point and multiple imputation methods will be used. Sensitivity analysis will be run on the number of intervention sessions received.

### Plans to give access to the full protocol, participant-level data, and statistical code {31c}

In line with the requirements of many peer-reviewed journals, de-identified participant-level data and the associated statistical code used for analyses will be made available upon reasonable request after the study results are published.

## Oversight and monitoring

### Composition of the coordinating centre and trial steering committee {5d}

No independent oversight committees will be convened. The study chief investigator (CI) (DC) and principal investigator (ASG) from the coordinating centre (UCL) will oversee the trial’s day-to-day activities, the adherence to the protocol and procedures for consenting, and ensure adequate data quality. The CI will inform the sponsor (UCL) should they have concerns which have arisen from monitoring activities, and/or if there are problems with oversight/monitoring procedures. The trial steering committee will meet regularly (every 6 months) to monitor progress and address any concerns that arise during the study. The committee will oversee scientific and ethical matters and ensure the trial stays aligned with the protocol.

### Composition of the data monitoring committee, its role and reporting structure {21a}

The trial manager (GG) in conjunction with the trial statistician (RE) will maintain ongoing data quality checks throughout the lifecycle of the project with oversight from lead statistician (ZH), ensuring any data queries are resolved quickly. The trial steering committee will meet regularly to ensure the ongoing data quality and conduct of the trial is maintained throughout. This process is conducted independently of the trial sponsor.

### Adverse event reporting and harms {22}

There are no major disadvantages or risks associated with participation. Each adverse event will be assessed for severity, causality, seriousness, and expectedness. All serious adverse events will be recorded in the medical records, the Clinical Research File (CRF), and the sponsor’s adverse event (AE) log, and will be monitored by the trial sponsor. The assessment of the relationship of adverse events to the procedure is a clinical decision based on all available information when the case report form is completed. The evaluation of the relationship of an adverse event to this/these additional safety issues will also be carried out as part of the study.

### Frequency and plans for auditing trial conduct {23}

The CI will ensure adequate quality and number of monitoring activities conducted by the study team. This will include adherence to the protocol and procedures for consenting and ensuring adequate data quality. The CI will inform the sponsor should they have concerns which have arisen from monitoring activities, and/or if there are problems with oversight/monitoring procedures.

### Plans for communicating important protocol amendments to relevant parties (e.g. trial participants, ethical committees) {25}

All protocol changes (e.g. amendments to the procedure, eligibility, outcomes, analyses) will be submitted to and require approval from the East of England—Essex Research Ethics Committee.

### Dissemination plans {31a}

Study results will be published in peer-reviewed academic journals and presented at public and scientific conferences. This work will also be highlighted on various (social) media channels.

## Discussion

Cognitive impairment in post-acute sequelae of SARS-CoV-2 infection (PASC) represents a newly acquired cognitive disorder, high in prevalence worldwide and associated with major morbidity in the absence of any established treatments. Cognitive impairment may occur even after mild COVID-19 infection and impacts patients’ daily function and quality of life to variable degrees. To address the need for an effective treatment, this randomised controlled trial will determine whether cognitive rehabilitation techniques, used effectively in other acquired cognitive disorders, may improve functional outcomes in people with cognitive impairment in PASC. A successful trial outcome will have major implications for the management of PASC and the quality of life of those affected by this condition.

## Trial status

Trial ongoing (Protocol V1.11 from 1^st^ May 2024, number EDGE 143067; IRAS project ID 302920). The recruitment for the RCT began on 13^th^ February 2023 and was completed on 6^th^ March 2024. The RCT intervention and data collection began in March 2023 and will be completed by 1^st^ July 2024. RCT will terminate in September 2024.

## Data Availability

Access to the final de-identified trial dataset will be restricted to specific members of the study team (CI, PI, and designated data analysts). There are no contractual agreements that limit access to the trial dataset within the study team. Upon the publication of the study results, the de-identified data may be shared publicly, as required by journals if all confidentiality and ethical guidelines are met.

## Abbreviations

BGSI: Bangor Goal-Setting Interview
CSRI: Client Service Receipt Inventory
CONSORT 2010: Consolidated Standards of Reporting Trials
D-KEFS: Delis–Kaplan Executive Functioning System
DSQ-PEM: DePaul Symptom Questionnaire—Post-Exertional Malaise
GAD-7: Generalised Anxiety Disorder Assessment
GRIPP2-SF: Guidance for Reporting Involvement of Patients and the Public
MS: Multiple sclerosis
IADL: Instrumental Activities of Daily Living
ICF: Informed Consent Form
LCCAB: Long COVID Cognitive Assessment Battery
PASC: Post-acute sequelae of SARS-CoV-2
PPI: Patient and Public Involvement
PCR: Polymerase chain reaction
PHQ-8: Patient Health Questionnaire
PSQI: Pittsburgh Sleep Quality
RCT: Randomised controlled trial
RBANS: Repeatable Battery for the Assessment of Neuropsychological Status
SF-DEM: Social Functioning in Dementia Scale
TBI: Traumatic brain injury
TOPF: Test of Premorbid Functioning
TAU: Treatment as usual
